# Methanol-Assisted CO_2_ Fixation by Hydroxyl-Containing Amidine Leading to Polymeric Ionic Liquid and Cross-Linked Network Formation

**DOI:** 10.3390/polym17243306

**Published:** 2025-12-14

**Authors:** Irina Irgibaeva, Nikolay Barashkov, Farkhad Tarikhov, Anuar Aldongarov, Lyazat Salkeeva, Gulzhian Dzhardimalieva, Yerbolat Tashenov

**Affiliations:** 1 Department of Chemistry, L.N. Gumilyov Eurasian National University, 2 Satpayev Str., 010008 Astana, Kazakhstan; irgibayeva_is@enu.kz; 2 Micro-Tracers, Inc., 1370 Van Dyke Avenue, San Francisco, CA 94124, USA; 3 Office of Research Core Facilities and HPC, National Laboratory Astana, Nazarbayev University, 53 Kabanbay Batyr Ave., 010000 Astana, Kazakhstan; 4 Department of Technical Physics, L.N. Gumilyov Eurasian National University, 2 Satpayev Str., 010008 Astana, Kazakhstan; 5 Department of Organic Chemistry and Polymers, Ye.A. Buketov Karaganda National Research University, 28 Universitetskaya Str., 100028 Karaganda, Kazakhstan; 6 Federal Research Center of Problems of Chemical Physics and Medicinal Chemistry, Russian Academy of Sciences, 1 Acad. Semenov Ave., 142432 Chernogolovka, Moscow Region, Russia; dzhardim@icp.ac.ru

**Keywords:** hydroxyl-containing amidine, methanol-assisted CO_2_ fixation, polymeric ionic liquid (PIL), cross-linked ionic polymer (CL-IP), hydrogen bonding, DFT calculations, FTIR and NMR spectroscopy, sustainable polymeric materials

## Abstract

This study presents a methanol-assisted pathway that converts hydroxyl-containing amidine into a polymeric ionic liquid (PIL) through direct CO_2_ fixation, followed by its transformation into a cross-linked ionic polymer (CL-IP). Methanol plays a crucial role in this process, acting as both a structural and electronic mediator. Its strong hydrogen-bonding interactions with amidine activate the molecule toward CO_2_ capture and promote the formation of ionic intermediates. Spectroscopic analyses (FTIR, ^1^H and ^13^C NMR) revealed the emergence of amidinium and alkyl-carbonate groups, while viscosity and mass measurements indicated progressive polymerization during CO_2_ absorption. Density functional theory calculations confirmed the stabilizing effect of methanol and the reduced HOMO–LUMO gap, which facilitates PIL formation. The subsequent condensation of the PIL with glutaraldehyde produced a dense three-dimensional cross-linked network (CL-IP), as verified by FTIR, XPS, SEM, and TGA analyses. These results highlight a straightforward and sustainable strategy for constructing hydrogen-bond-mediated ionic polymers capable of tunable CO_2_ capture and potential application in environmentally compatible materials.

## 1. Introduction

Ionic liquids (ILs) constitute a broad class of organic salts that remain liquid over extended temperature ranges and are characterized by negligible vapor pressure, high thermal and chemical stability, and tunable physicochemical properties. Recent definitions no longer rely on the conventional melting point criterion of 100 °C, instead emphasizing structural and functional features that enable ion-dominated behavior in condensed phases [1]. Owing to these characteristics, ILs are widely investigated as functional media and active components in catalysis, electrochemistry, separation processes, and advanced energy-conversion systems [1,2,3,4].

Among various IL families, amidinium-based systems attract considerable attention due to their strong Brønsted basicity, ability to form stable ion pairs, and versatile coordination behavior. Several studies have reported the preparation of mono- and dicationic amidinium salts with diverse structural motifs and demonstrated their applicability as electrolytes, catalysts, and functional reaction media [5,6,7,8,9,10,11]. However, despite their potential, amidinium ILs remain significantly less explored than other protic ionic liquids, partly due to the limited commercial availability of amidine precursors and the structural diversity arising from hydrogen bonding networks [12,13,14]. As a result, systematic relationships between structure, reactivity, and physicochemical behavior in amidinium systems remain insufficiently understood.

Polymeric ionic liquids (PILs), in which ionic fragments are incorporated into macromolecular backbones, expand the functionality of ILs by combining ionic conductivity with enhanced mechanical and thermal stability [15,16,17,18]. Designing PILs from highly basic amidines is of particular interest because amidines can readily interact with electrophiles and acidic species, enabling reactive routes to polymeric materials under mild conditions [19]. In this context, CO_2_-responsive amidines represent a promising platform for generating PIL-type structures through direct reaction with carbon dioxide.

The interaction of amidines with CO_2_ has been extensively studied and is known to proceed through multiple pathways depending on the molecular environment, proton donors, and hydrogen-bonding patterns. Recent work by Morselli et al. demonstrated that even for strongly basic amidines such as DBU, the outcome of CO_2_ fixation is highly sensitive to the presence of protic impurities and the ionic environment, leading to the formation of zwitterionic CO_2_ adducts rather than simple bicarbonate or carbamate species [20]. In the presence of alcohols, water, or other protic species, the reaction may involve formation of carbamate, bicarbonate, or alkyl-carbonate species, accompanied by protonation of the amidine to yield amidinium salts [21,22,23,24,25,26]. The competition between these pathways is influenced by the availability of nucleophilic nitrogen centers, the presence of hydrogen-bond donors, and solvation effects. As demonstrated in several studies, protic solvents can modulate the accessibility of the amidine lone pair and stabilize different CO_2_-derived anions, thereby altering the outcome of CO_2_ fixation [23,24,25,26,27]. Consequently, elucidating the role of the reaction medium is essential for understanding and controlling PIL formation based on amidines.

Despite the growing body of work on protic ILs and CO_2_-responsive organic bases, relatively little is known about how hydroxyl-containing amidines behave during CO_2_ addition and how protic solvents influence the composition and structure of the resulting ionic products. Previous reports have shown that condensation of N,N-dimethylformamide dimethyl acetal with alkanolamines yields amidines capable of undergoing CO_2_ capture and subsequent polymerization under mild conditions [27,28]. However, the mechanistic basis for the formation of polymeric ionic liquids from such systems, particularly the role of methanol in stabilizing intermediates and directing the reaction pathway, remains insufficiently clarified.

The present study addresses this gap by examining the CO_2_ fixation behavior of a hydroxyl-functionalized amidine prepared from tris(hydroxymethyl)aminomethane and N,N-dimethylformamide dimethyl acetal. Particular attention is given to comparing the reactions carried out in the presence and absence of methanol, with the aim of identifying how methanol affects the structure of the CO_2_-derived ionic species and the subsequent formation of polymeric ILs. Using FTIR, NMR, thermal analysis, and DFT calculations, we analyze the structural features of the obtained materials and assess the mechanistic implications of methanol-assisted stabilization. Furthermore, the ability of the resulting polymeric ionic liquid to undergo glutaraldehyde-mediated cross-linking is evaluated to demonstrate the formation of a cross-linked ionic polymer network.

## 2. Materials and Methods

### 2.1. Chemicals

All reagents employed in this work were of analytical grade and were used as received without any additional purification. The synthesis of amidines was carried out using tris(hydroxymethyl)aminomethane (C_4_H_11_NO_3_, purity ≥ 99.9%, biochemical grade, Sigma-Aldrich, St. Louis, MO, USA) and N,N-dimethylformamide dimethyl acetal (C_5_H_13_NO_2_, purity ≥ 97%, Thermo Fisher Scientific, Waltham, MA, USA). For the preparation of the polymeric ionic liquid, high-purity carbon dioxide gas (99.9%) was utilized. The cross-linking step involved a 25% aqueous solution of glutaraldehyde (C_5_H_8_O_2_, Sigma-Aldrich, St. Louis, MO, USA) in the presence of phosphoric acid (H_3_PO_4_, purity ≥ 99.0%, Sigma-Aldrich, St. Louis, MO, USA), which served as a catalyst. In addition, isopropyl alcohol (C_3_H_8_O, purity ≥99.5%, Sigma-Aldrich, St. Louis, MO, USA) was used as the reference solvent in viscosity measurements.

### 2.2. Synthesis

#### 2.2.1. Preparation of Amidine (1a–b) with/Without Methanol Removal

Method 1. Amidine (1a) synthesis with methanol removal has been performed following procedure published earlier [27]: The amidine (1a) was prepared in a three necked 100 mL round-bottom flask equipped with a magnetic stirrer, a reflux condenser, and a Dean–Stark apparatus. Dimethyl formamide dimethyl acetal (12.0 g, 0.099 mol) was introduced and heated to 50 °C in a water bath. Under constant stirring, tris(hydroxymethyl)aminomethane (11.8 g, 0.099 mol) gradually added in small portions. The next portion was added only after complete dissolution of the previous one. During the reaction, the methanol formed as a by-product was continuously removed through the Dean–Stark trap. After complete addition, the reaction mixture was maintained at 50 °C for 3 h, then the temperature increased to 60 °C and stirring continued for an additional 30 min. The product (1a) was obtained as a viscous, light-yellow liquid. The yield, based on the volume of methanol collected, was 97%.

^1^H NMR (ppm, CDCl_3_, 500 MHz) δ: 3.33 (s, 6H, –N(CH_3_)_2_), 3.52 (dd, J = 9.94, 11.47 Hz, 6H, –CH_2_–OH), 4.11 (s, 3H, –OH), 6.87 (s, 1H, =CH–N).

^13^C NMR (ppm, CDCl_3_, 125 MHz) δ: 38.62 (–N(CH_3_)_2_), 64.91 (–CH_2_–OH), 75.56 (C_q_), 156.88 (=CH–N).

FTIR (cm^−1^): 3278 (br, νO–H), 2927, 2834 (ν_as_, ν_s_CH_3_/CH_2_), 1625 (νC=N), 1459–1389 (δCH_2_, δC–OH), 1097, 1023, 934 (νC–O, νC–N).

Method 2. Amidine (1b) synthesis without methanol removal: In a separate series of experiments, the reaction was carried out under similar conditions but without methanol removal. Dimethylformamide dimethyl acetal (12.0 g, 0.099 mol) was placed in a 100 mL round-bottom flask fitted with a magnetic stirrer and a reflux condenser. The solution was heated to 50 °C, and tris(hydroxymethyl)aminomethane (11.8 g, 0.099 mol) was added in small increments, each portion being introduced only after the previous one had completely dissolved. The mixture was stirred at 50 °C for 3 h, followed by heating at 60 °C for another 30 min. In this case, the methanol generated during the reaction was not removed but retained within the system. The resulting product (1b) having light-yellow color was less viscous than product (1a).

^1^H NMR (ppm, CDCl_3_, 500 MHz) δ: 2.37 (s, 1H, MeOH), 2.85 (s, 3H, –N(CH_3_)_2_), 2.93 (s, 3H, –N(CH_3_)_2_), 3.42 (s, 3H, MeOH), 3.59 (dd, J = 11.51, 18.40 Hz, 6H, –CH_2_–OH), 4.15 (s, 2H, –OH), 6.91 (s, 1H, =CH–N), 7.97 (s, 1H, –OH, H-bonded to MeOH).

^13^C NMR (ppm, CDCl_3_, 125 MHz) δ: 38.62 (–N(CH_3_)_2_), 64.91 (–CH_2_–OH), 70.73 (MeOH), 75.56 (C_q_), 156.88 (=CH–N).

FTIR (cm^−1^): 3308 (br, νO–H, hydrogen-bonded), 2934, 2829 (ν_as_, ν_s_CH_3_/CH_2_), 1628 (νC=N), 1460–1370 (δCH_2_, δC–OH), 1096, 1023, 934 (νC–O, νC–N).

#### 2.2.2. Conversion of Amidine into Polymeric Ionic Liquids (PILs)

The conversion of amidine (1b) into a PIL (2) was performed at 25 °C by bubbling carbon dioxide (CO_2_) through the amidine solution. The CO_2_ flow rate was adjusted so that approximately 3–4 bubbles per second passed through the liquid phase.

The duration of CO_2_ bubbling ranged from 20 to 80 min. During this time, no precipitation was observed, and the solutions remained transparent; however, a noticeable increase in viscosity was recorded as the reaction progressed. The extent of CO_2_ uptake was monitored gravimetrically by weighing the vessel containing amidine before and after the introduction of CO_2_. Measurements were taken every 20 min throughout the 80 min bubbling process.

^1^H NMR (ppm, CDCl_3_, 500 MHz) δ: 2.35 (s, 1H), 2.75 (s, 2H), 3.05 (s, 1H), 3.30 (s, 4H), 3.50 (s, 4H), 3.60 (s, 2H), 4.07 (s, 2H), 5.13 (s, 2H), 6.87 (s, 1H).

^13^C NMR (ppm, CDCl_3_, 125 MHz) δ: 36.72, 37.46, 50.01, 62.35, 64.18, 64.38, 70.86, 75.40, 77.44, 155.34, 156.71, 162.87, 164.06.

FTIR (cm^−1^): 3257 (br, νO–H, νN–H^+^), 2927, 2834 (ν_as_, ν_s_CH_3_/CH_2_), 1698, 1662 (νC=O, νC=N), 1536–1440 (δCH_3_, wCH_2_), 1258–1026 (νC–O, νC–N).

#### 2.2.3. Viscosity Measurements

The relative viscosity of amidine (1b) and PIL samples (20–80 min CO_2_ treatment) was determined using a Cannon–Fenske capillary viscometer at room temperature. Isopropyl alcohol was employed as the reference liquid. For each measurement, the efflux time of the sample was recorded in triplicate at room temperature, and the average value was used for calculations. The relative viscosity (η_rel_) was calculated as the ratio of the flow time of the sample to that of the reference solvent.

#### 2.2.4. Preparation of Cross-Linked Ionic Polymer (CL-IP, 3)

Cross-linked ionic polymer (3) was synthesized starting from the PIL (2) (18.0 g). The polymer (2) was mixed with 180 g of a 25% aqueous glutaraldehyde solution. Concentrated orthophosphoric acid was added as a catalyst at a dosage of 2–3 drops per 100 mL of solution. The reaction mixture was stirred continuously at 50 °C for 1 h.

During the reaction, a foamy, yellowish material was formed, corresponding to the cross-linked ionic polymer (3). The product (3) was collected by filtration, thoroughly washed with distilled water, and subsequently freeze-dried at −50 °C for 24 h. The dried material (3) was obtained as a powder with a characteristic pale-yellow color. The final product (3) was stored at −4 °C until further characterization.

FTIR (cm^−1^): 3349 (br, νO–H), 2927, 2869 (νC–H), 1717, 1686 (νC=O, νC=N overlapped), 1453, 1374 (δC–N, δC–O), 1115, 1042, 986 (νC–O–C, νN–C–O).

### 2.3. Characterization

#### 2.3.1. FTIR Characterization

Fourier transform infrared spectroscopy (FTIR) was employed to examine the characteristic functional groups of amidines (1a-b), PILs 40–60–80 (2), and CL-IP (3). Spectra were recorded using a Nicolet™ 6700 FTIR spectrometer (Thermo Scientific, Madison, WI, USA) fitted with a diamond attenuated total reflectance (ATR) accessory. Each sample was analyzed directly in ATR mode without additional preparation. The spectra were acquired in the range of 4000–500 cm^−1^, with a spectral resolution of 4 cm^−1^, and 32 individual scans were averaged for each run to improve the signal-to-noise ratio. The raw data was processed and interpreted using Omnic 5.2 software.

#### 2.3.2. Nuclear Magnetic Resonance (NMR) Spectroscopy

NMR spectra of amidines (1a–b) and PIL (2) samples (PIL-40, PIL-60, PIL-80) were recorded using a JNM-ECA 500 FT-NMR spectrometer (JEOL, Japan, Tokyo, Akishima) equipped with a compensated self-shielded solenoid magnet (500 MHz for ^1^H, 125 MHz for ^13^C). Measurements were carried out in deuterated chloroform (CDCl_3_) as solvent. The number of scans was optimized to ensure adequate signal-to-noise ratio.

#### 2.3.3. Thermogravimetric and Differential Scanning Calorimetry (TGA-DSC)

Thermal stability of the synthesized PIL (2) and CL-IP (3) samples was examined by TGA-DSC using a STA 449 F3 Jupiter instrument (NETZSCH, Selb, Germany). Approximately 30 mg of each sample was weighed and placed in an aluminum oxide crucible. The measurements were carried out under an inert argon atmosphere to prevent oxidative degradation. The temperature range was set from 25 °C to 90 °C, with a heating rate of 5 °C min^−1^ for PIL (2), and from 30 °C to 1250 °C, with a heating rate of 10 °C min^−1^ for CL-IP (3).

#### 2.3.4. Scanning Electron Microscopy (SEM)

The morphology and surface characteristics of the cross-linked ionic polymer (3) were investigated using scanning electron microscopy (SEM). Measurements were carried out on a JEOL (JEOL JSM-IT800, Akishima, Tokyo, Japan) system operating at an accelerating voltage of 5.0 kV, with a working distance of 4.2 mm. The microscope was run in secondary electron detection (SED) mode. Samples were analyzed in the range of 1000–50,000 magnifications. The scale bars varied from 10 μm down to 100 nm.

#### 2.3.5. X-Ray Photoelectron Spectroscopy (XPS)

X-ray photoelectron spectroscopy (XPS) analysis for CL-IP (3) was carried out using a Thermo Scientific Nexsa system (Madison, WI, USA), operating with a monochromatic Al Kα radiation source (1486.6 eV). The instrument was configured in constant analyzer energy (CAE) mode with standard lens operation. A survey spectrum was first collected at a pass energy of 100 eV (step size 1.0 eV, 1361 steps, two scans, acquisition time 27.2 s to identify the elemental composition. High-resolution scans were subsequently recorded for the C 1s (pass energy 50 eV, step size 0.1 eV, 241 steps, five scans, total time 60.3 s), N 1s (pass energy 50 eV, 0.1 eV step size, 231 steps, six scans, total time 69.3 s), and O 1s (pass energy 50 eV, 0.1 eV step size, 231 steps, three scans, total time 34.7 s) regions. The X-ray spot size was maintained at 400 μm throughout all measurements. Charge compensation was applied using the system’s flood gun. Spectral fitting and deconvolution were performed with OriginPro-2024 software.

### 2.4. Computational Study

Quantum-chemical calculations of the molecular structure and properties of the investigated systems were performed using the density functional theory (DFT) method with the three-parameter hybrid exchange–correlation functional Becke–Lee–Yang–Parr (B3LYP) for closed-shell systems. The split-valence basis set 6-31G(d,p), supplemented with polarization functions d on heavy atoms and p on hydrogen atoms was employed [29].

Full geometry optimization was carried out for all considered systems without imposing symmetry constraints, regardless of the local symmetry of alkyl substituents. Solvent effects were considered at the B3LYP/6-31G(d,p) level using the polarizable continuum model (IEFPCM). To account for dispersion interactions in complex formation, the DFT-D3 approach was applied. All computations were performed using the Gaussian 16, Revision A.03 software package [30].

## 3. Results

### 3.1. Synthesis of Amidines (1a–b) and Their Characterization

**Figure 1:**
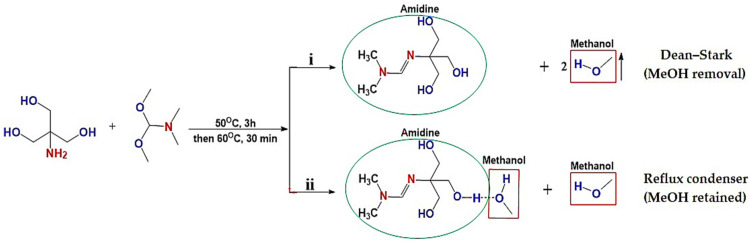
Synthetic schemes for amidines (1a–b): (**i**) Reaction with continuous removal of methanol using a Dean–Stark trap, leading to amidine (1a); (**ii**) Reaction under reflux condenser without methanol removal, resulting in amidine (1b) stabilized by hydrogen-bonded methanol.

The amidine compounds were successfully synthesized via the condensation of tris(hydroxymethyl)aminomethane with N,N-dimethylformamide dimethyl acetal under reflux conditions (Figure 1) [27]. Two approaches were investigated: (i) amidine (1a) obtained with continuous removal of methanol as a by-product, and (ii) amidine (1b) formed in the presence of methanol retained in the system. In both cases, the reactions proceeded smoothly, affording viscous light-yellow liquids in high yields (>95%). The key difference between the two methods lies in the role of methanol, which in the second case remained in solution and formed hydrogen-bonded complexes with amidine, thus influencing the physicochemical properties.

The structures of amidines (1a–b) were confirmed by proton and carbon NMR spectroscopy (Table S1). The ^1^H and ^13^C NMR spectra of amidines (1a–b) recorded in CDCl_3_ reveal distinct differences depending on whether methanol is removed during synthesis or retained in the system. In the methanol-free sample, the –N(CH_3_)_2_ protons appear as a singlet at δ 3.33 ppm, while in the methanol-containing sample they split into two distinct singlets at δ 2.85 and 2.93 ppm, accompanied by a corresponding shift in the carbon signal at δ 38.62 ppm. The hydroxyl signals also undergo changes: the broad singlet at δ 4.11 ppm (OH) becomes δ 4.15 ppm, and an additional downfield resonance at δ 7.97 ppm appears, consistent with a hydrogen-bonded OH group. Furthermore, the methanol CH_3_ resonance is observed at δ 3.42 ppm in the proton spectrum and δ 70.73 ppm in the carbon spectrum. These spectral changes clearly indicate that methanol participates in hydrogen bonding with amidine, influencing both proton and carbon environments [31,32,33,34]. The complete spectra are provided in the Supporting Information (Table S1 and Figures S1–S4).

### 3.2. Conversion of Amidines into Polymeric Ionic Liquids (PILs)

The reactivity of amidines toward CO_2_ strongly depended on the presence of methanol in the system [35]. When the methanol-free compound (1a) was exposed to a CO_2_ stream, no detectable changes in mass, viscosity, or spectroscopic features were observed, indicating the absence of further transformation. In contrast, the amidine–MeOH complex (1b) displayed a markedly different behavior.

**Figure 2:**
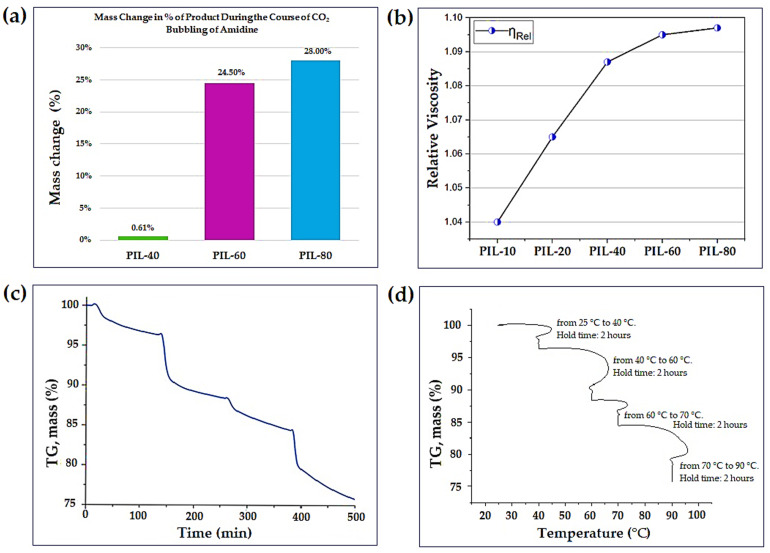
(**a**) Mass change (%) of the product during CO_2_ bubbling of amidine (1b) at different reaction times (40–80 min). (**b**) Variation of relative viscosity (η_rel_) of PILs derived from amidine (1b) as a function of CO_2_ bubbling time (10–80 min). (**c**) TGA curve of PIL (2) showing the time-dependent mass change during heating from 25 to 90 °C. (**d**) Temperature-dependent representation of TGA profile of PIL (2) obtained using a stepwise heating program.

During CO_2_ bubbling, the solutions of (1b) remained optically clear but gradually increased in viscosity. Gravimetric monitoring showed that the mass of the mixture was essentially unchanged during the first 40 min, but then rose sharply between 40 and 60 min, consistent with the onset of CO_2_ uptake. By 80 min, the mass gain reached approximately 28%, suggesting near saturation of amidine with CO_2_ (Figure 2a) [36]. A similar trend was observed in viscosity measurements, where the relative viscosity increased from η_rel_ = 1.050 for the initial amidine to η_rel_ = 1.097 after 80 min (Figure 2b and Tables S2 and S3) [37]. These macroscopic observations provide the first indication that methanol plays a key role in enabling the interaction of amidine (1b) with CO_2_.

**Figure 3:**
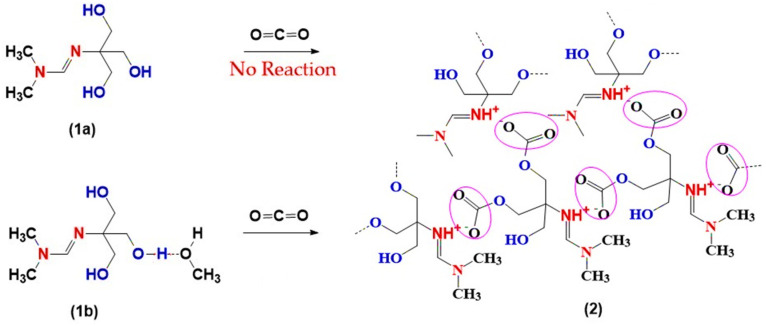
Reactions of amidines (1a–b) with CO_2_: effect of methanol.

This contrasting outcome is summarized in Figure 3. In the absence of methanol, amidine (1a) remains unreactive toward CO_2_. In sharp contrast, the methanol-containing system (1b) undergoes efficient CO_2_ incorporation, giving rise to polymeric ionic liquid structures (2).

Thermogravimetric analysis (TGA) of PIL (2) was carried out under an argon atmosphere. The data from one experiment are shown in two forms (Figure 2c,d). In the time-based curve (Figure 2c); the mass remains essentially unchanged up to about 40 °C. Further heating leads to several distinct mass-loss steps, and by 90 °C the sample retains about 75.8% of its initial mass. The observed decrease (~24.2%) corresponds to CO_2_-released from the material.

The same dataset is plotted against temperature in Figure 2d, which highlights the programmed heating segments used during the experiment (25–40 °C, 40–60 °C, 60–70 °C and 70–90 °C). The mass-loss steps seen at these temperatures correspond to the changes in Figure 2c. The release of CO_2_ occurs gradually across these intervals, which is consistent with the reversible binding of CO_2_ in PIL (2) [38].

#### Spectroscopic Evidence of PIL (2) Formation

**Figure 4:**
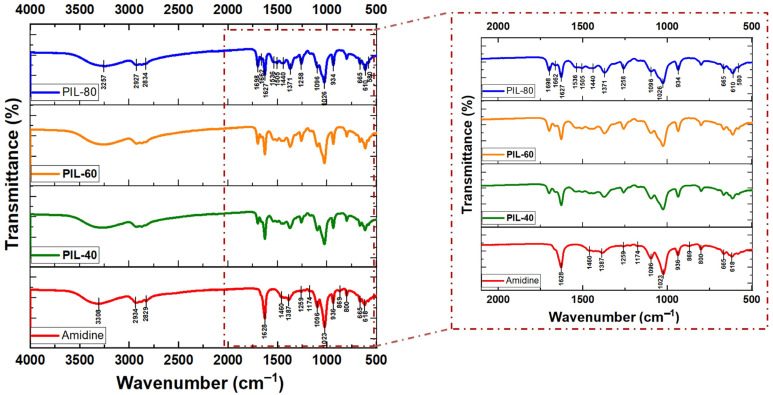
FTIR spectra of amidine–MeOH (1b) and the corresponding PILs obtained after CO_2_ bubbling for 40, 60, and 80 min.

The conversion of amidine (1b) into PILs upon exposure to CO_2_ was monitored using FTIR spectroscopy in combination with NMR and viscosity measurements. The FTIR spectra of amidine–MeOH complex (1b) and the resulting PILs obtained at different CO_2_ bubbling times (40, 60, and 80 min) are shown in Figure 4, while the main band assignments are summarized in Table S4.

In the spectrum of amidine (1b), the characteristic absorption of the C=N bond was observed at 1628 cm^−1^, along with broad O–H stretching bands around 3308 cm^−1^. Upon CO_2_ incorporation, new bands appeared at 1662 cm^−1^ and 1698 cm^−1^, attributable to protonated amidinium (–CH=N^+^H–) and alkyl-carbonate groups, respectively. These features became more intense with increasing reaction time, consistent with the progressive transformation of amidine (1b) into PIL (2). Additional absorptions at 1258–1440 cm^−1^ and 1026–1096 cm^−1^correspond to –C–O stretching vibrations, further confirming CO_2_ fixation [39].

For comparison, the FTIR spectra of amidine (1a) (without methanol) before and after CO_2_ bubbling showed no noticeable changes in either the C=N or O–H regions, confirming that no PIL (2) formation occurs under these conditions (Figure S5). This highlights the crucial role of methanol in stabilizing the amidine intermediate and enabling CO_2_ incorporation.

**Figure 5:**
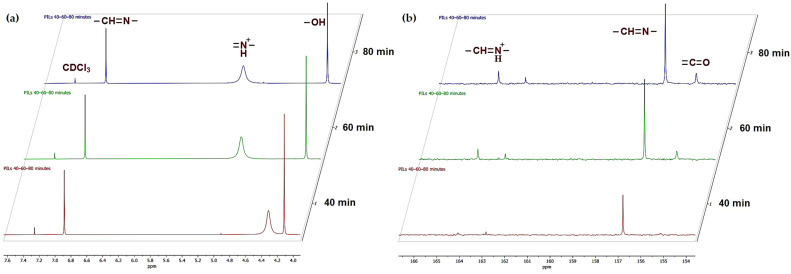
NMR monitoring of amidine–MeOH complex during CO_2_ bubbling: (**a**) ^1^H NMR spectra and (**b**) ^13^C NMR spectra recorded after 40, 60, and 80 min, demonstrating progressive conversion to PILs.

The structural evolution of amidine–MeOH complex (1b) upon CO_2_ bubbling was further monitored by NMR spectroscopy. The ^1^H NMR spectra recorded after 40, 60, and 80 min (Figure 5a) reveals distinct changes in the chemical environment of the amidine protons. The initial spectrum is dominated by the resonance of the amidine proton (–CH=N–) at δ = 6.87 ppm. With increasing reaction time, a new signal emerges at δ ≈ 4.25–5.13 ppm, which can be attributed to the protonated amidinium species (–CH=N^+^H–), indicating the progressive incorporation of CO_2_, consistent with enhanced hydrogen bonding interactions in the developing PIL (2) network [40,41].

The ^13^C NMR spectrum (Figure 5b) further supports this transformation. The resonance of the amidine carbon is observed at δ = 156.88 ppm in the starting molecule (1b). After CO_2_ exposure, an additional signal appears at δ ≈ 155–156 ppm, which is assigned to alkyl-carbonate carbon atoms, indicating CO_2_ fixation. Moreover, the amidinium carbon resonance (–CH=N^+^H–) becomes evident around δ ≈ 164–165 ppm, with intensity increasing from 40 to 80 min, correlating with the progressive growth of the polymeric ionic liquid structure [40,41].

By contrast, the methanol-free amidine (1a) showed none of these spectral changes under identical conditions, further suggesting its inability to generate PILs. The corresponding proton and carbon NMR spectra are provided in the Supporting Information (Figure S6a,b).

Overall, the NMR results clearly demonstrate that amidine in the presence of methanol undergoes a gradual transformation into PILs upon CO_2_ uptake, in full agreement with the IR data and viscosity/mass change analyses.

### 3.3. Computational Insights into Methanol-Assisted PIL Formation

To rationalize the experimental findings, the Density Functional Theory (DFT) calculations at the B3LYP/6-31G(d,p) level were carried out to compare the stability of amidine both in the absence and presence of methanol. The solvent effect of methanol was considered through the Integral Equation Formalism Polarizable Continuum Model (IEFPCM) with a dielectric constant corresponding to methanol. Comparison of the electronic energies revealed a notable stabilization of the system in the methanol environment (∆E = 19.504 kcal·mol^−1^) [42]:

E (amidine 1a) = −800.105334 a.u.

E (amidine 1b) = −800.136418 a.u.

**Figure 6:**
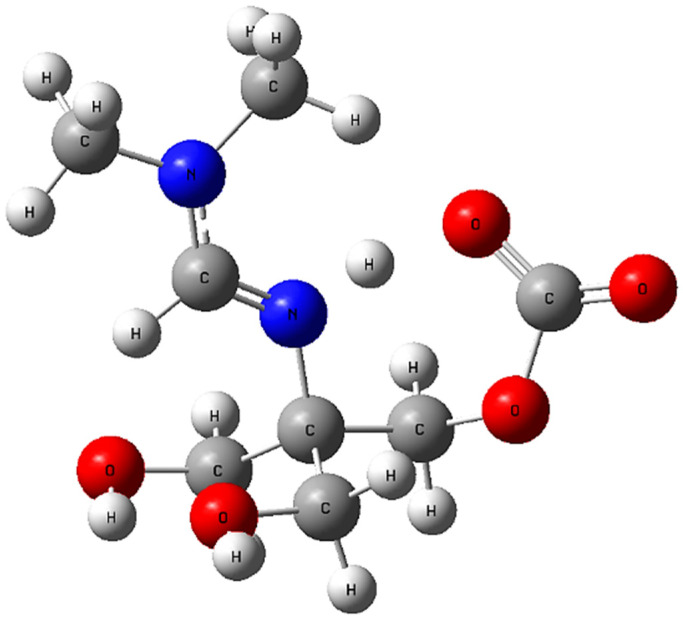
Optimized geometry of the amidine–CO_2_ zwitterionic adduct calculated with implicit methanol solvation (IEFPCM). Atom colors: C (grey), H (white), O (red), N (blue).

This energy decrease clearly indicates that methanol provides a thermodynamically more favorable baseline for subsequent reactions. In other words, the presence of methanol lowers the overall energy of the amidine system and thereby facilitates the subsequent interaction with CO_2_ leading to the zwitterionic PIL (2) structure (Figure 6).

**Figure 7:**
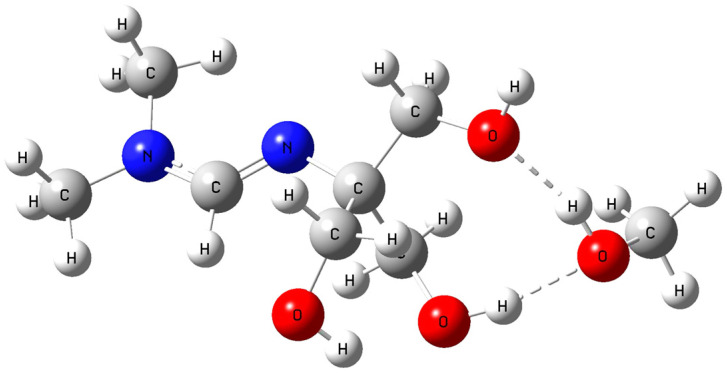
Optimized geometry of the amidine–MeOH (1b) complex showing intermolecular hydrogen bonding. Atom colors: C (grey), H (white), O (red), N (blue).

**Figure 8:**
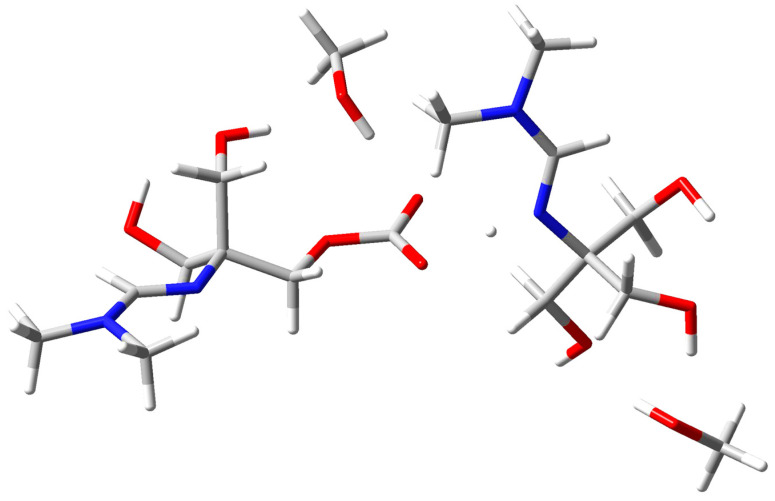
Optimized geometry of the amidine–MeOH–CO_2_ complex stabilized by multiple hydrogen bonds. Atom colors: C (grey), H (white), O (red), N (blue).

The optimized structure of amidine–MeOH (1b) revealed two strong intermolecular O–H···O hydrogen bonds (1.780 Å and 1.784 Å) and an additional intramolecular contact (1.986 Å), forming a preorganized H–bonding scaffold (Figure 7). Upon introduction of CO_2_, this adduct evolved into the amidine–MeOH–CO_2_ complex, where multiple O–H···O/N interactions (1.685–2.003 Å) and an N···H contact further stabilized the assembly (Figure 8) [43].

This transformation was accompanied by an increase in dipole moment (from ~4.1 D in amidine–MeOH (1b) to ~6.5 D in amidine–MeOH–CO_2_), a lowering of the LUMO energy, and narrowing of the HOMO–LUMO gap. The calculated structural and electronic parameters for the amidine–MeOH (1b) and amidine–MeOH–CO_2_ complexes are listed in Table 1. These electronic signatures reflect enhanced charge delocalization and polarizability, consistent with the experimental observation that methanol enables CO_2_ capture and subsequent PIL formation.

In conclusion, these results demonstrate that methanol not only stabilizes the amidine energetically but also preorganizes it electronically and structurally to favor the binding of CO_2_, thereby explaining why PIL (2) forms exclusively in the presence of methanol.

### 3.4. Synthesis and Characterization of Cross-Linked Ionic Polymer (CL-IP)

**Figure 9:**
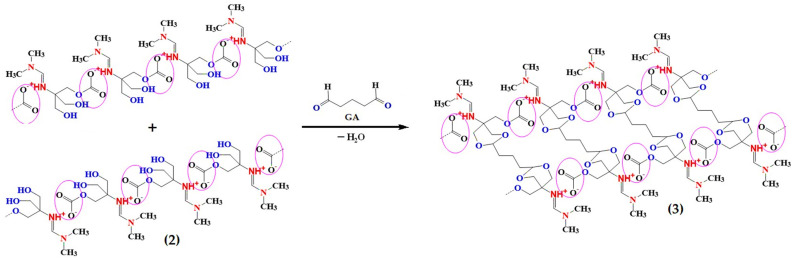
Schematic representation of the cross-linking reaction between PIL (2) and glutaraldehyde (GA) forming CL-IP (3) through condensation.

The CL-IP (3) was synthesized from PIL (2) through glutaraldehyde-mediated condensation [44] in the presence of phosphoric acid, yielding a pale-yellow foamy material (Figure 9). The formation of covalent cross-links between hydroxyl and aldehyde groups was confirmed by FTIR, XPS, SEM, and TGA analyses.

**Figure 10:**
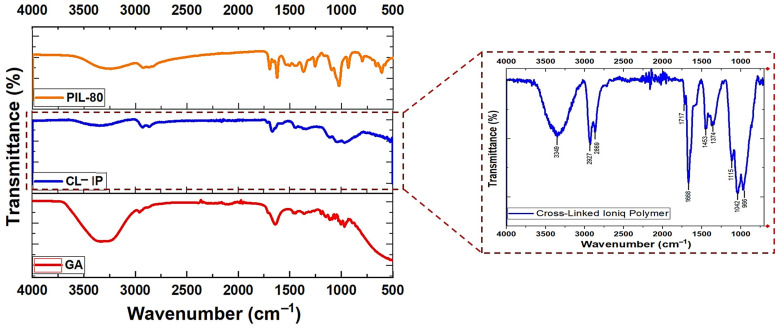
FTIR spectra of PIL (2), CL-IP (3), and GA.

The FTIR spectra of glutaraldehyde (GA), PIL (2), and CL-IP (3) are shown in Figure 10. The spectrum of GA displays characteristic absorptions at 1717 cm^−1^(C=O stretching of the aldehyde group), 2960 cm^−1^ and 2877 cm^−1^ corresponding to C–H stretching vibrations. After cross-linking with PIL (2), several notable spectral changes confirm the formation of the new CL-IP (3) network.

A broad band centered at 3349 cm^−1^ is attributed to O–H stretching vibrations, arising from residual hydroxyl. The bands near 2927 cm^−1^ and 2869 cm^−1^ correspond to aliphatic C–H stretching modes of methylene groups. The disappearance of the free aldehyde C=O band at 1717 cm^−1^ indicates the consumption of the aldehyde groups. The reaction of GA with the hydroxyl groups of PIL (2) leads to the formation of acetal linkages, which do not contain a C=O group. Therefore, the remaining absorption near 1686 cm^−1^ originates primarily from C=N stretching of the amidinium moieties. The absorptions at 1453 cm^−1^ and 1374 cm^−1^ are assigned to C–N and C–O bending vibrations. In the fingerprint region, the bands at 1115, 1042 and 986 cm^−1^ correspond to C–O–C stretching modes characteristic of acetal linkages, confirming the formation of GA-derived cross-links, together with N–C–O modes originating from PIL (2) [45].

As a whole, the disappearance of the aldehyde C=O band and the growth of the C–O–C region provides clear evidence for acetal formation between GA and PIL (2), confirming the successful generation of the cross-linked polymer (3).

**Figure 11:**
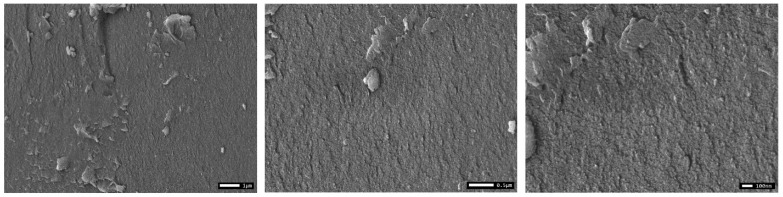
SEM micrographs of CL-IP (3) at different magnifications.

The surface morphology of CL-IP (3) was examined using scanning electron microscopy (Figure 11). The SEM images reveal a relatively homogeneous and compact morphology with minor surface irregularities, indicating the formation of a dense polymeric network after cross-linking with glutaraldehyde. The surface of CL-IP (3) appears rougher and more consolidated, which is consistent with the formation of cross-linking bridges within the polymer matrix. The absence of distinct crystalline domains further supports the amorphous nature of the resulting material.

**Figure 12:**
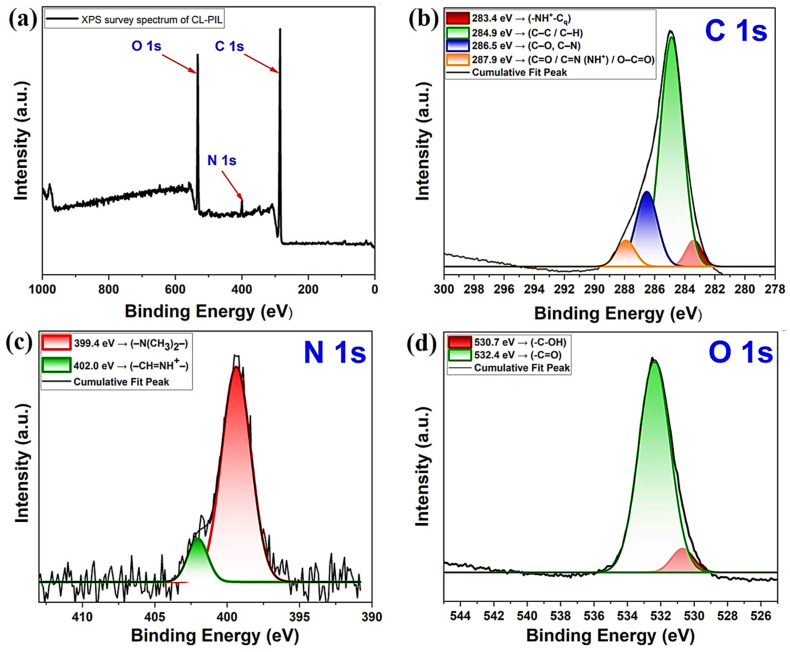
(**a**) XPS survey scan and high resolution XPS spectra of (**b**) C 1s, (**c**) N 1s, and (**d**) O 1s for CL-IP (3).

The XPS survey spectrum of CL-IP (3) confirmed the presence of carbon, nitrogen, and oxygen as the main elements (Figure 12a). The dominant C 1s peak was observed at ~285 eV, accompanied by characteristic contributions from N 1s (~399–402 eV) and O 1s (~531–532 eV).

High-resolution analysis of the C 1s region revealed four components (Figure 12b): a peak at 283.4 eV assigned to quaternized carbon adjacent to protonated nitrogen (–NH^+^–Cq), a strong contribution at 284.9 eV from aliphatic C–C/C–H bonds, a component at 286.5 eV corresponding to C–O and C–N groups, and a higher-binding energy peak at 287.9 eV related to carbonyl, amidine (–CH=N and –CH=NH^+^), and carboxyl species [46,47].

The N 1s spectrum (Figure 12c) displayed two well-defined peaks. The major contribution at 399.4 eV was attributed to tertiary amine nitrogen (–N(CH_3_)_2_–), whereas a second peak at 402.0 eV originated from amidinium species (–CH=NH^+^–) [48].

The O 1s spectrum (Figure 12d) exhibited two main components: the peak at 530.7 eV corresponded to hydroxyl and/or acetal oxygen (–C–OH/–C–O–C), while the signal at 532.4 eV was assigned to carbonyl oxygen (–C=O) [49].

**Figure 13:**
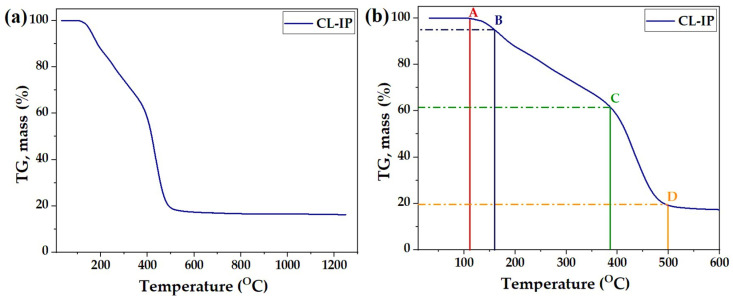
TGA curve of CL-IP (3): (**a**) full TGA profile recorded in the temperature range of 30–1250 °C; (**b**) magnified section up to 600 °C. Points A–D indicate key thermal events: A (113 °C) – onset of minor weight loss (moisture/solvent); B (160 °C) – initial structural rearrangement; C (386 °C) – major degradation of organic linkages; D (500 °C) – final weight-loss step leading to carbonaceous residue.

The thermal stability of CL-IP (3) was evaluated by thermogravimetric analysis (TGA) under an argon atmosphere. The TGA curve (Figure 13a) shows a multi-step weight loss behavior over the temperature range of 30–1250 °C. The polymer exhibits high thermal stability up to approximately 100 °C, indicating the absence of physically adsorbed volatile impurities.

The enlarged section of the TGA curve up to 600 °C (Figure 13b) highlights four main transition points. Point A (113 °C) corresponds to the onset of minor weight loss associated with adsorbed moisture and residual solvent, with 100% of the initial mass retained. Point B (160 °C) shows the beginning of structural rearrangement and the initial cleavage of weak hydrogen-bonded interactions, resulting in a mass retention of 95%. A more pronounced degradation step is observed at Point C (386 °C), where approximately 62% of the sample mass remains, attributed to the decomposition of organic linkages within the polymeric network. The final major weight loss occurs at Point D (500 °C), leaving about 19% of the residue, likely corresponding to the formation of a stable carbonaceous chain.

In summary, the TGA profile indicates that CL-IP (3) possesses excellent thermal stability, with significant degradation only beginning above 350 °C, confirming the robust nature of the cross-linked ionic polymer (3).

## 4. Discussion

The collective experimental and theoretical findings clearly demonstrate that methanol plays a decisive role in activating hydroxyl-containing amidine toward CO_2_ fixation and the subsequent formation of PILs. In the absence of methanol, amidine (1a) remains chemically inert, showing no signs of CO_2_ uptake. In contrast, in the methanol-containing medium (1b), a distinct transformation occurs—methanol interacts through multiple hydrogen bonds with the hydroxyl and –C=N functionalities of amidine, forming a stabilized intermediate complex that serves as a precursor to zwitterionic PIL structures. This dual experimental–computational insight indicates that methanol functions not merely as a solvent but as a structural and electronic mediator fundamentally influencing the reactivity of amidine toward CO_2_ [35,37].

Spectroscopic analyses provide compelling evidence supporting this mechanism. The ^1^H NMR spectra of the methanol-containing amidine (1b) displays characteristic resonances of hydrogen-bonded methanol molecules, including a singlet near 3.42 ppm corresponding to the CH_3_ group of methanol and a broad signal around 7.97 ppm associated with the hydrogen-bonded hydroxyl proton of amidine (1b). In the parent amidine, the –N(CH_3_)_2_ protons give a single singlet at 3.33 ppm. In the methanol-containing form (1b), this signal splits into two singlets at 2.85 and 2.93 ppm. Such splitting is best explained by a partial restriction of rotation around the C–N bond, which makes the two methyl groups non-equivalent. Similar effects have been reported for guanidinium ionic liquids, where hydrogen-bonded species create an asymmetric environment near the nitrogen center and slow down internal rotation [50]. In our case, the methanol molecule interacts with the amidine through a stable hydrogen-bond contact. This interaction likely positions methanol near one side of the –N(CH_3_)_2_group and leads to the observed splitting.

Upon introducing CO_2_ into the sample, several new resonances appear in the ^13^C NMR spectrum of PIL (2), mainly in the region of 155–165 ppm. The signal at 155.34 ppm can be assigned to the carbonyl carbon of the alkyl-carbonate group that forms after CO_2_ addition. The peaks at 156.7 and 164.1 ppm arise from the –C=N fragments of the amidinium unit and reflect protonation at the nitrogen, respectively. Further information comes from the behavior of the –CH_2_–O groups. In the parent amidine (1b), this carbon resonates at 64.91 ppm, while in PIL (2) it appears at 62.35, 64.18, and 64.38 ppm. Jessop and co-workers showed that in systems where alcohols and DBU bind CO_2_, the –CH_2_–O resonance usually shifts slightly downfield upon formation of the [ROCO_2_][DBUH] adduct [51]. A similar trend is seen here, although the changes are smaller. This is expected, because in PIL (2) each –CH_2_–O group lies between the positively charged amidinium fragment and the CO_2_-derived group, both of which withdraw electron density. Such an arrangement can counteract the downfield shift typically observed for simple alcohol– CO_2_ adducts and explains why the methylene carbon signals remain in the 62–65 ppm range (Figure S8). Although the carbonyl region alone does not allow a definitive structural assignment, the appearance of a carbonyl signal associated with a –CH_2_–O fragment at about 155 ppm, together with the modest but systematic changes in the –CH_2_–O region, is most consistent with the formation of an alkyl-carbonate species in PIL (2).

These spectroscopic findings correlate with macroscopic observations, including a rise in relative viscosity from η_rel_ = 1.050 to 1.097 and a total mass gain of about 28% after 80 min of CO_2_ absorption [36,39]. The simultaneous increase in mass and viscosity provides strong evidence of methanol-assisted CO_2_ fixation.

An FTIR spectrum further substantiates these conclusions. The pristine amidine exhibits a C=N stretching band at 1628 cm^−1^, whereas the spectrum of PIL-80 features new absorptions between 1698 cm^−1^ and 1662 cm^−1^, attributable to overlapping C=O and –CH=NH^+^ vibrations of alkyl-carbonate and amidinium moieties. The broad O–H stretching band shifts from 3308 cm^−1^ to 3257 cm^−1^, reflecting stronger hydrogen-bonding interactions within the polymeric ionic framework. These spectral transformations unequivocally confirm the incorporation of CO_2_ into the methanol-stabilized amidine, yielding zwitterionic structures of the –CH=NH^+^···^−^O–COO– type. This observation agrees with previous studies on CO_2_-responsive amine, squaramide and imidazole systems, where hydrogen-bond donors enhance CO_2_ uptake efficiency by stabilizing intermediate species [52,53,54,55,56]. However, in the present system, methanol not only facilitates solubility but also directly participates in electronic stabilization and charge delocalization, leading to a unique methanol-mediated polymeric ionic structure.

TGA supports the dynamic nature of this process. The PIL (2) sample remains stable up to approximately 40 °C, followed by a gradual ~24.2% mass loss between 40 °C and 90 °C due to reversible CO_2_ release. This reversible weight loss demonstrates that the ionic linkages formed during CO_2_ absorption are not permanent and can dissociate under mild heating, enabling potential cyclic CO_2_ capture and release, which is characteristic of weak, reversible alkyl-carbonate formation rather than formation of thermally stable carbamate species. Comparable thermal reversibility has been reported for ammonium- and imidazolium-based PILs, confirming that weakly bound CO_2_ species can desorb below 100 °C through similar chemisorption–desorption equilibria [57]. Such reversibility highlights the tunable, dynamic character of the methanol-assisted PIL formation process.

DFT calculations provide molecular-level confirmation of these findings. At the B3LYP/6-31G(d,p) level with solvent correction via the Integral Equation Formalism Polarizable Continuum Model (IEFPCM), solvation in methanol lowers the total electronic energy of amidine by approximately 19.5 kcal·mol^−1^ compared to the gas-phase structure. The optimized geometry reveals two strong O–H···O hydrogen bonds (1.78 Å) between methanol and amidine, which stabilize the molecule and align reactive centers favorably for CO_2_ binding. Upon CO_2_ coordination, multiple O–H···O/N hydrogen bonds (1.685–2.003 Å) form, increasing the dipole moment from 4.125 D to 6.485 D and narrowing the HOMO–LUMO gap. These effects enhance charge polarization and electron delocalization, which rationalizes the experimentally observed increase in reactivity [42,58]. The DFT results therefore confirm that methanol stabilizes the transition states and intermediates, lowering the energy barrier for CO_2_ activation and promoting ionic bond formation within the PIL matrix.

Subsequent cross-linking of PIL (2) with glutaraldehyde (GA) produced CL-IP (3) with improved mechanical and thermal stability. The FTIR spectrum of the cross-linked material shows the complete disappearance of the aldehyde C=O band of GA at 1717 cm^−1^ and the emergence of intensified C–O–C stretching bands in the 1115–986 cm^−1^ region, which are characteristic of acetal linkages formed through the condensation of aldehydes with hydroxyl groups. The remaining absorption near 1686 cm^−1^ originates from the C=N stretching of the amidinium units. These demonstrate the successful formation of covalent linkages. SEM images show a compact, uniform morphology with a slightly roughened texture, suggesting denser cross-linking and reduced porosity. This morphological evolution parallels other PIL-based hydrogels and ion-conductive polymers, where covalent cross-linking enhances rigidity and dimensional stability [45,59].

Beyond their structural and mechanistic significance, these results also reveal the practical potential of the synthesized materials. The cross-linked polymer (3) exhibits high stability up to 160 °C and partial biodegradability, making it a promising component for eco-friendly polymeric matrices or hydroponic substrates [44,60]. Such materials could be reused or naturally degraded after cultivation, minimizing environmental impact and aligning with sustainable development principles. Hence, the obtained PIL (2) and CL-IP (3) systems combine CO_2_-responsiveness with environmental compatibility, opening prospects for green polymer design, gas capture, and agricultural applications.

In summary, this work establishes a clear methanol-assisted pathway for the transformation of amidines into polymeric ionic liquids and their cross-linked derivatives. Methanol acts as both a structural and electronic promoter, enabling CO_2_ activation through hydrogen-bond-mediated stabilization. The resulting materials demonstrate reversible CO_2_ binding, strong ionic character, and tunable structural integrity. These findings expand the understanding of hydrogen-bond-assisted CO_2_ fixation mechanisms and provide a foundation for designing new classes of responsive, environmentally benign polymeric materials [37,61].

## 5. Conclusions and Future Perspectives

In the present study, a simple and effective approach for carbon dioxide utilization was developed based on the reaction of tris(hydroxymethyl)aminomethane with N,N-dimethylformamide dimethyl acetal to obtain a hydroxyl-containing amidine. This amidine served as a precursor for PIL formation through methanol-assisted CO_2_ fixation. The process is attractive due to its mild conditions and the ability of methanol to facilitate both structural organization and electronic activation via hydrogen bonding. The experimental results, supported by DFT modeling, confirmed the conversion of the amidine into ionic species containing amidinium and carbonate/carboxylate groups. The obtained PIL exhibited reversible CO_2_ binding, with partial gas release occurring upon mild heating, which demonstrates its dynamic and regenerable character.

The cross-linked ionic polymer obtained by condensation of the PIL with glutaraldehyde exhibited enhanced thermal stability relative to the non-cross-linked PIL. FTIR, XPS, SEM, and TGA analyses confirmed the formation of a dense three-dimensional polymeric framework. This stability is associated with the firm fixation of alkyl-carbonate fragments within the cross-linked structure, underscoring the potential of this material as a thermally robust CO_2_-derived polymeric system.

In future work, the biodegradability and environmental behavior of the CL-IP will be evaluated to explore its applicability as a component of polymeric materials for hydroponic cultivation and other sustainable uses. The development of eco-compatible polymeric networks capable of partial degradation after their service life could offer a green alternative to conventional substrates. Further investigations will focus on understanding the degradation kinetics, nutrient sorption, and physicochemical parameters of these materials under controlled hydroponic and soil-free conditions. Such studies may pave the way toward integrating CO_2_-derived polymers into environmentally responsible agricultural and material technologies.

## Figures and Tables

**Table 1:** Optimized structural and electronic parameters of amidine–MeOH (1b) and amidine–MeOH–CO_2_ complexes calculated at the B3LYP/6-31G(d,p) level.

Model	Key H-Bonds (Å)	Dipole μ (D)	E_HOMO_ (a.u.)	E_LUMO_ (a.u.)
Amidine–MeOH (1b)	Intermol. O–H···O: 1.780 and 1.784 Intramol. O–H···O: 1.986	4.125	−0.2008	+0.0346
Amidine–MeOH–CO_2_	O–H···O/N: 1.685–2.003	6.485	−0.1878	−0.0265

HOMO—Highest Occupied Molecular Orbital; LUMO—Lowest Unoccupied Molecular Orbital; μ—dipole moment; Å—angstrom; D—Debye; a.u.—atomic units.

## Data Availability

The original contributions presented in this study are included in the article/Supplementary Material. Further inquiries can be directed to the corresponding author.

## Supplementary Materials

The following supporting information can be downloaded at: https://www.mdpi.com/article/10.3390/polym17243306/s1, Figure S1: ^1^H NMR spectrum of amidine (1a) in CDCl_3_ (500 MHz, without methanol); Figure S2: ^1^H NMR spectrum of amidine (1b) in CDCl_3_ (500 MHz, with methanol); Figure S3: ^13^C NMR spectrum of amidine (1a) in CDCl_3_ (125 MHz, without methanol); Figure S4: ^13^C NMR spectrum of amidine (1b) in CDCl_3_ (125 MHz, with methanol); Figure S5: FTIR spectra of amidine (1a) before and after CO_2_ bubbling, showing no significant changes; Figure S6: (a) ^1^H NMR spectra of amidine (without MeOH) after CO_2_ bubbling for 40, 60, and 80 min (recorded in CDCl_3_). (b) ^13^C NMR spectra of amidine (without MeOH) after CO_2_ bubbling for 40, 60, and 80 min (recorded in CDCl_3_); Figure S7: ^1^H NMR spectrum of PIL-80 (2) recorded in CDCl_3_; Figure S8: ^13^C NMR spectrum of PIL-80 (2) recorded in CDCl_3_; Table S1: Key ^1^H and ^13^C NMR chemical shifts (δ, ppm) of amidines (1a–b) with and without methanol (CDCl_3_, 500 MHz for ^1^H, 125 MHz for ^13^C); Table S2: Results of efflux time measurements for amidine (1b) and PIL samples; Table S3: Relative viscosity (η_rel_) of amidine (1b) and PILs at different CO_2_ bubbling times; Table S4: Key IR bands (cm^−1^) and assignments for amidine (1b) vs. PIL (2).
