# Supplementary material for: Methanol-Assisted CO2 Fixation by Hydroxyl-Containing Amidine Leading to Polymeric Ionic Liquid and Cross-Linked Network Formation
Source: Polymers (Basel). 2025 Dec 14;17(24):3306. doi: 10.3390/polym17243306 (PMC12736439; doi:10.3390/polym17243306)
Supplement: Supplementary file 1 [file polymers-17-03306-s001.zip › polymers-3956263-supplementary.pdf]

# Supplementary Materials

**Table S1.** Key $^1$H and $^{13}$C NMR chemical shifts (δ, ppm) of amidines (1a-b) with and without methanol ($CDCl_3$, 500 MHz for $^1$H, 125 MHz for $^{13}$C).

| Group / Assignment | Amidine (1a) (no MeOH) | | | Amidine (1b) (with MeOH) | | |
|---|---|---|---|---|---|---|
| | $^1$H NMR δ (ppm) | Multiplicity | $^{13}$C NMR δ (ppm) | $^1$H NMR δ (ppm) | Multiplicity | $^{13}$C NMR δ (ppm) |
| –$N(CH_3)_2$ (2 × $CH_3$) | 3.33 (6H) | s | 38.62 | 2.85, 2.93 (each 3H) | s, s | 38.62 |
| –$CH_2$–OH (3 × $CH_2$) | 3.52 (6H) | dd (J = 9.94, 11.47 Hz) | 64.91 | 3.59 (6H) | dd (J = 11.51, 18.40 Hz) | 64.91 |
| –OH (hydroxyl) | 4.11 (3H) | s | - | 4.15 (2H) | s | - |
| =CH–N (amidine proton) | 6.87 (1H) | s | 156.88 | 6.91 (1H) | s | 156.88 |
| –OH (H-bonded to MeOH) | - | - | - | 7.97 (1H) | s | - |
| – $CH_3$ (MeOH) | - | - | - | 3.42 (3H) | s | 70.73 |
| Quaternary C | - | - | 75.56 | - | - | 75.56 |

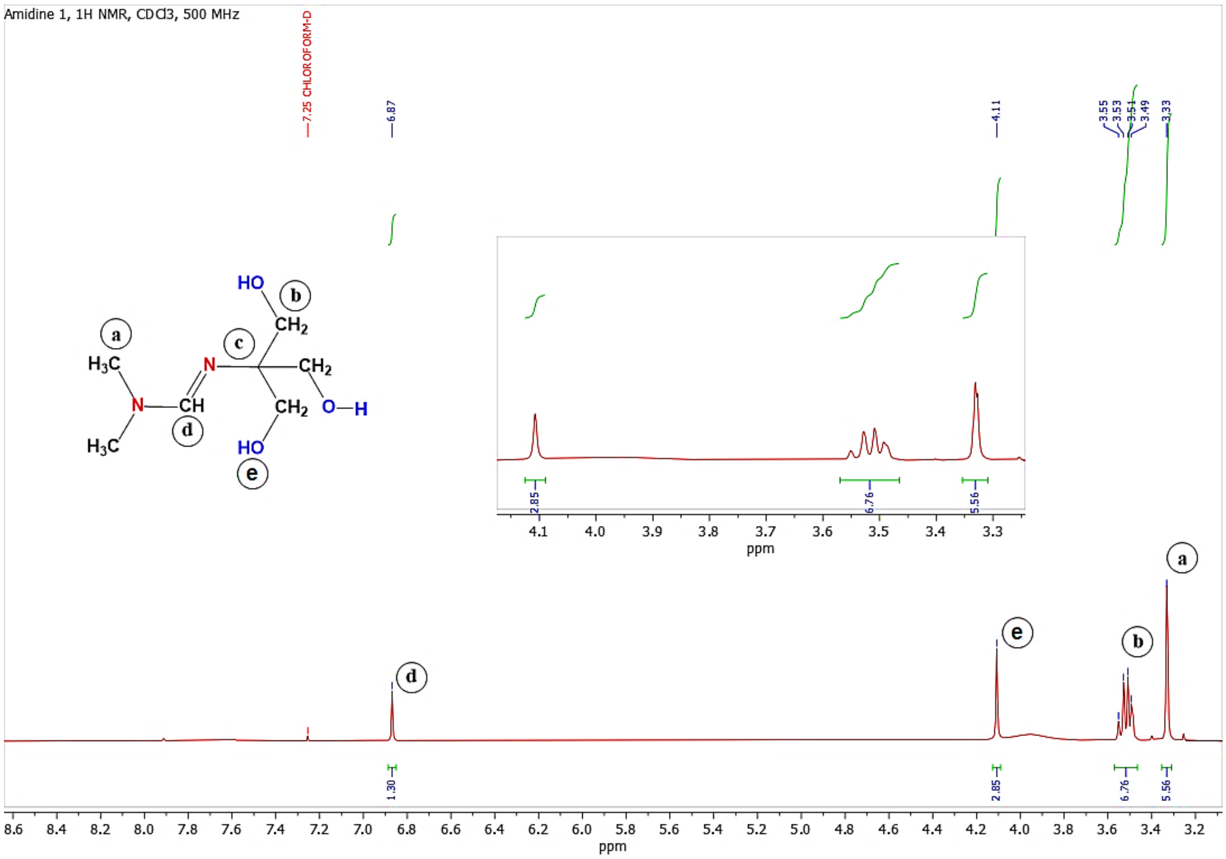


**Figure S1.**$^{1}$H NMR spectrum of amidine (1a) in $CDCl_3$ (500 MHz, without methanol).

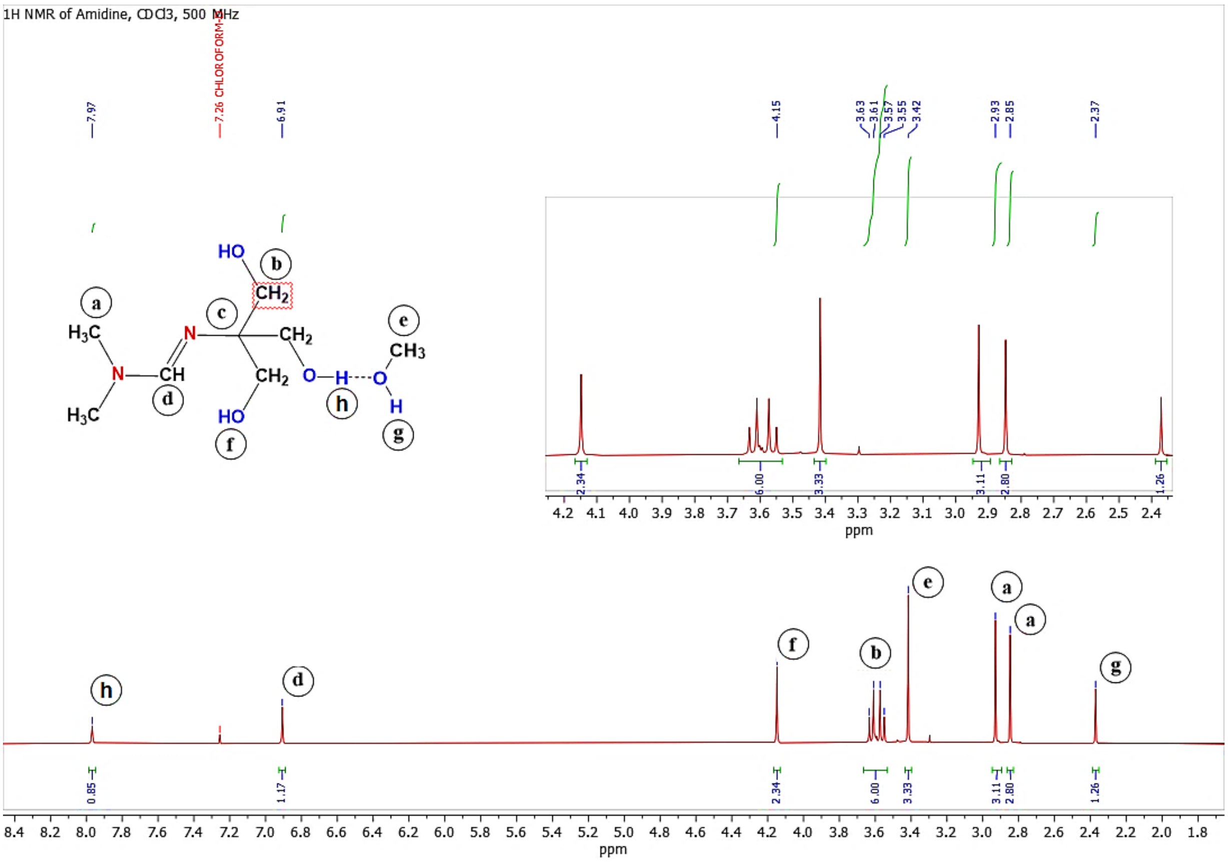


**Figure S2.**$^{1}$H NMR spectrum of amidine (1b) in $CDCl_3$ (500 MHz, with methanol).

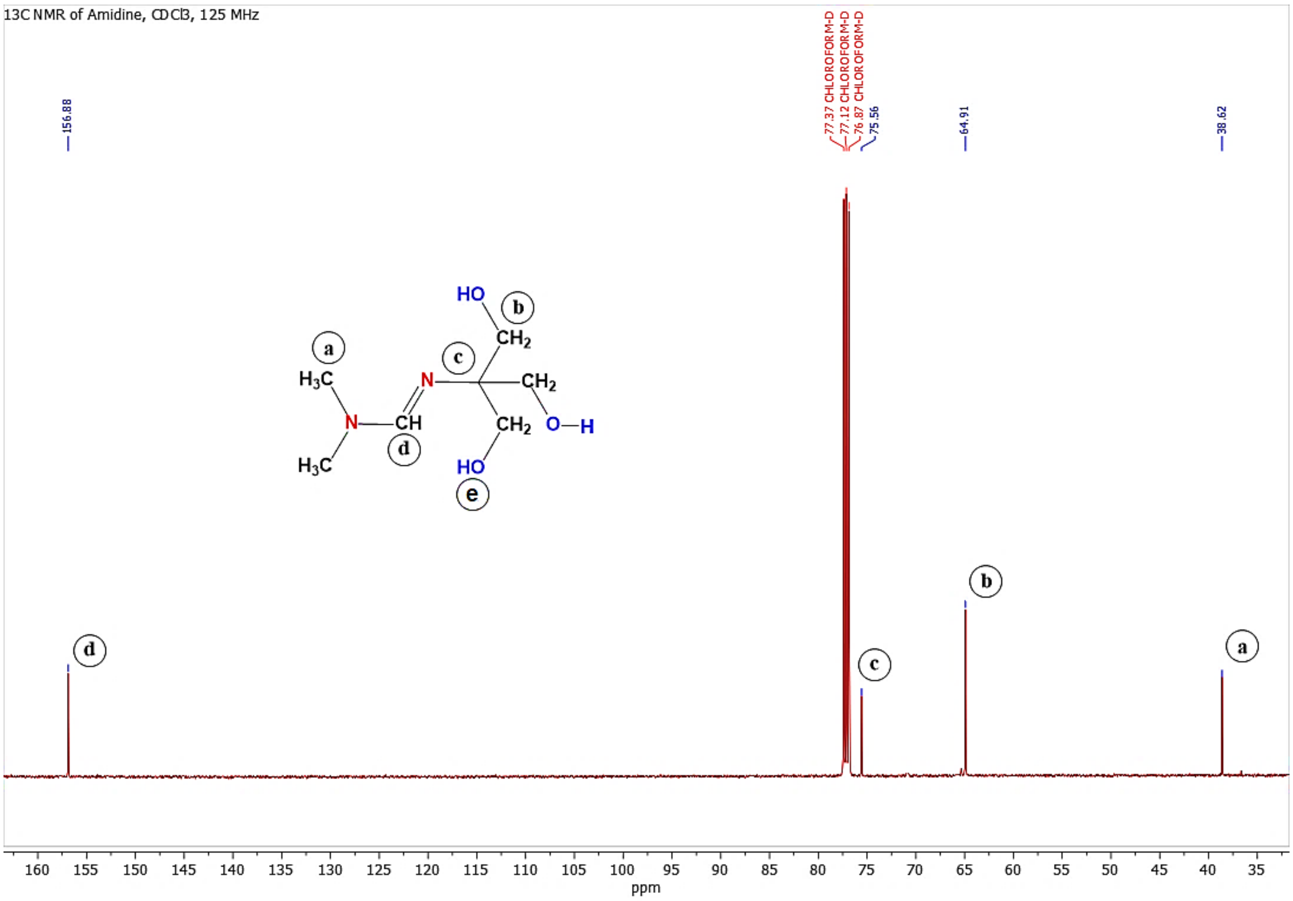


**Figure S3.**$^{13}$C NMR spectrum of amidine (1a) in $CDCl_3$ (125 MHz, without methanol).

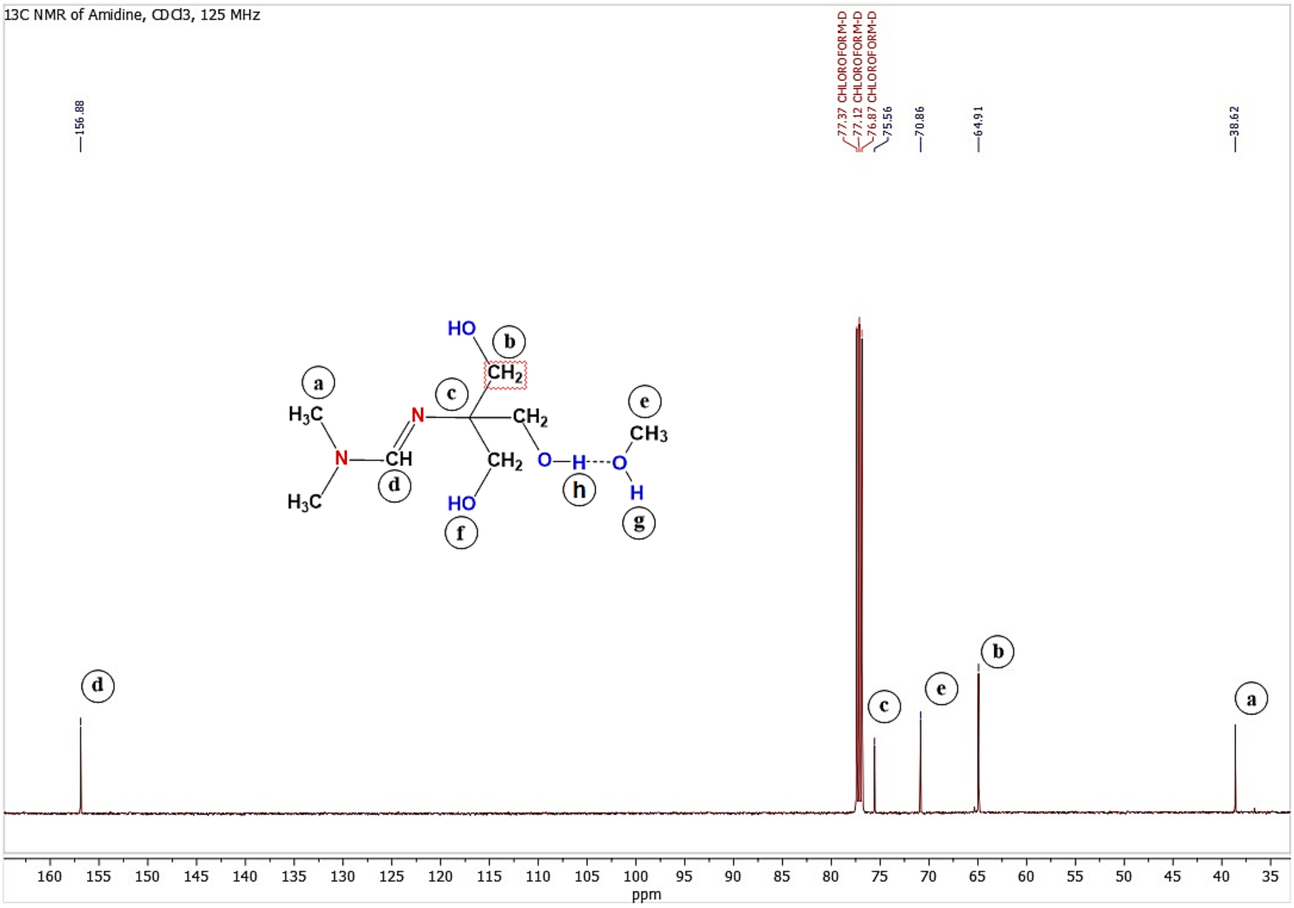


**Figure S4.**$^{13}$C NMR spectrum of amidine (1b) in $CDCl_3$ (125 MHz, with methanol).

**Table S2.** Results of efflux time measurements for amidine (1b) and PIL samples.

| Sample | ET-1, s | ET-2, s | ET-3, s | AET ± SD, s |
|---|---|---|---|---|
| i-Pr-OH (ref.) | 234 | 234 | 234 | 234.0 ± 0.00 |
| Amidine (1b) | 247 | 246 | 246 | 246.3 ± 0.58 |
| PIL-10 | 244 | 243 | 243 | 243.3 ± 0.58 |
| PIL-20 | 248 | 250 | 250 | 249.3± 1.15 |
| PIL-40 | 256 | 254 | 253 | 254.3 ± 1.53 |
| PIL-60 | 256 | 256 | 257 | 256.3 ± 0.58 |
| PIL-80 | 257 | 256 | 257 | 256.7 ± 0.58 |

*ET - Efflux Time; AET – Average Efflux Time; SD – Standard Deviation; i-Pr-OH – Isopropanol.

**Table S3.** Relative viscosity ($\eta_{Rel}$) of amidine (1b) and PILs at different $CO_2$ bubbling times.

| Sample | Relative viscosity $\eta_{rel}$ | $\eta_{rel}$ ± SD |
|---|---|---|
| Amidine (1b) | 1.050 | 1.050 ± 0.003 |
| PIL-10 | 1.040 | 1.040 ± 0.002 |
| PIL-20 | 1.065 | 1.065 ± 0.005 |
| PIL-40 | 1.087 | 1.087 ± 0.007 |
| PIL-60 | 1.095 | 1.095 ± 0.002 |
| PIL-80 | 1.097 | 1.097 ± 0.002 |

*SD – Standard Deviation.

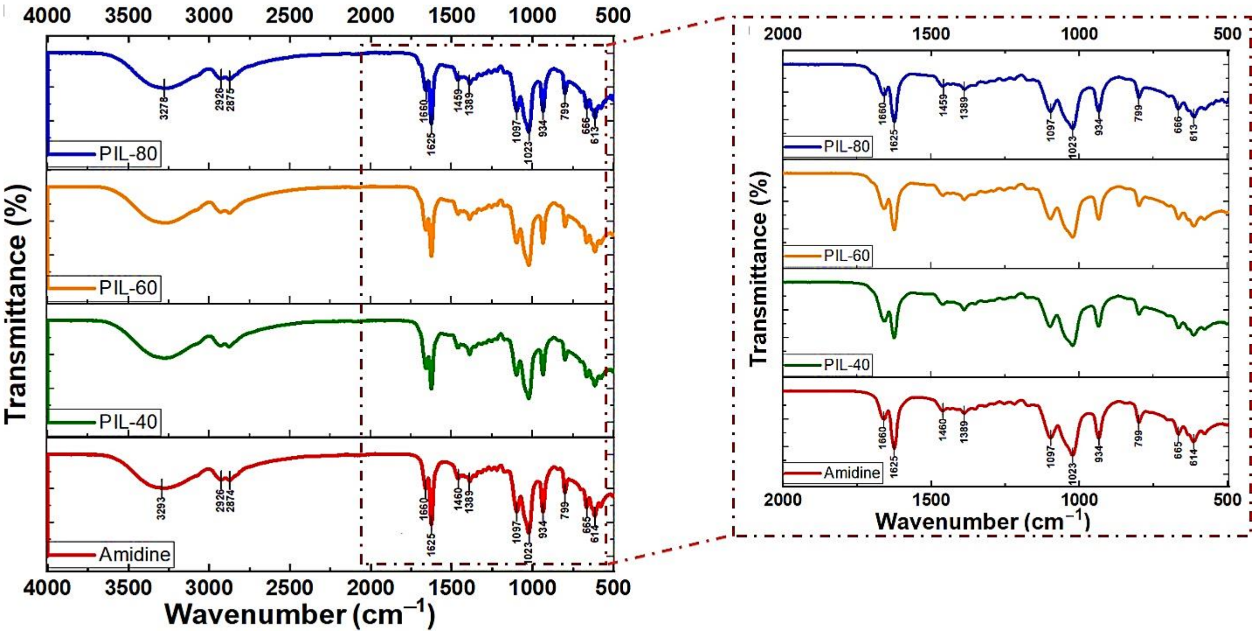


**Figure S5.** FT-IR spectra of amidine (1a) before and after $CO_2$ bubbling, showing no significant changes.

**Table S4.** Key IR bands ($cm^{-1}$) and assignments for amidine (1b) vs. PIL (2).

| Assignment (main contribution) | Amidine (1b) ν ($cm^{-1}$) | PIL (2) ν ($cm^{-1}$) | Comment |
|---|---|---|---|
| ν(O–H) stretch (H-bonded) | 3308 | 3257 | Red-shift on $CO_2$ uptake (stronger H-bonding) |
| $\nu(N\ldots H)^+$ (H-bonded/ion pair) | - | 3257 | Due to protonated N-center |
| $\nu_{as}$, $\nu_s(CH_3, CH_2)$ | 2934, 2829 | 2927, 2834 | Small intensity/position changes |
| ν(C=O) ($CO_2$-derived) | — | 1698 (s) | New diagnostic for PIL formation |
| $\nu(–CH=N^+H–)$ (protonated amidinium) | — | 1662 (sh) | Shoulder near 1698 $cm^{-1}$ |
| ν(C=N) | 1628 | 1627 | Persists with slight shift |
| $\delta(CH_3)$, $w(CH_2)$, $\tau(CH_2)$, δ(C-OH) | 1460 | 1536, 1505, 1440 | Intensity growth in PIL |
| mixed (ν(C–N), ϱ(CH), δ(OH)) | 1387 | 1371 | Amidine zone (partly shifts/splits) |
| $\nu(C(O_2)–O)$, $w(CH_2)$ | — | 1258 | $CO_2$-related skeletal/C–O |
| $\nu_{as}$, $\nu_s$(C–O) / ν(C–N) | 1096, 1023 (s) | 1096, 1026 (s) | Strengthening/splitting in PIL |
| $\nu((CH_2)–O))$, δ(C-OH), $\varrho(CH_2)$ | 936 | 934 | Moving into neighboring mixed region |
| ϱ(OH), $\nu_s$(C–C), δ(NCN) | 800 | 799 | Small shift upon PIL formation |
| δ(OCO), $\varrho(CH_2)$, δ(HCH), δ(NCN) | — | 799 | Bending of OCO fragment |

*ν – stretching; $\nu_{as}$ – asymmetric stretching; $\nu_s$ – symmetric stretching; δ – bending; γ – rocking; ϱ – wagging; τ – twisting.

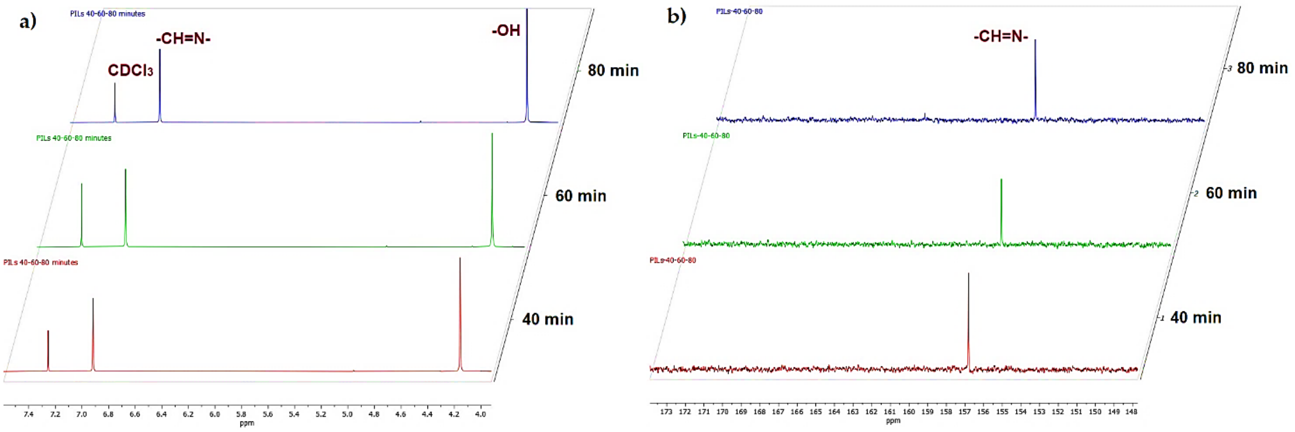


**Figure S6.**(**a**) $^{1}$H NMR spectra of amidine (without MeOH) after $CO_2$ bubbling for 40, 60, and 80 min (recorded in $CDCl_3$). (**b**) $^{13}$C NMR spectra of amidine (without MeOH) after $CO_2$ bubbling for 40, 60, and 80 min (recorded in $CDCl_3$).

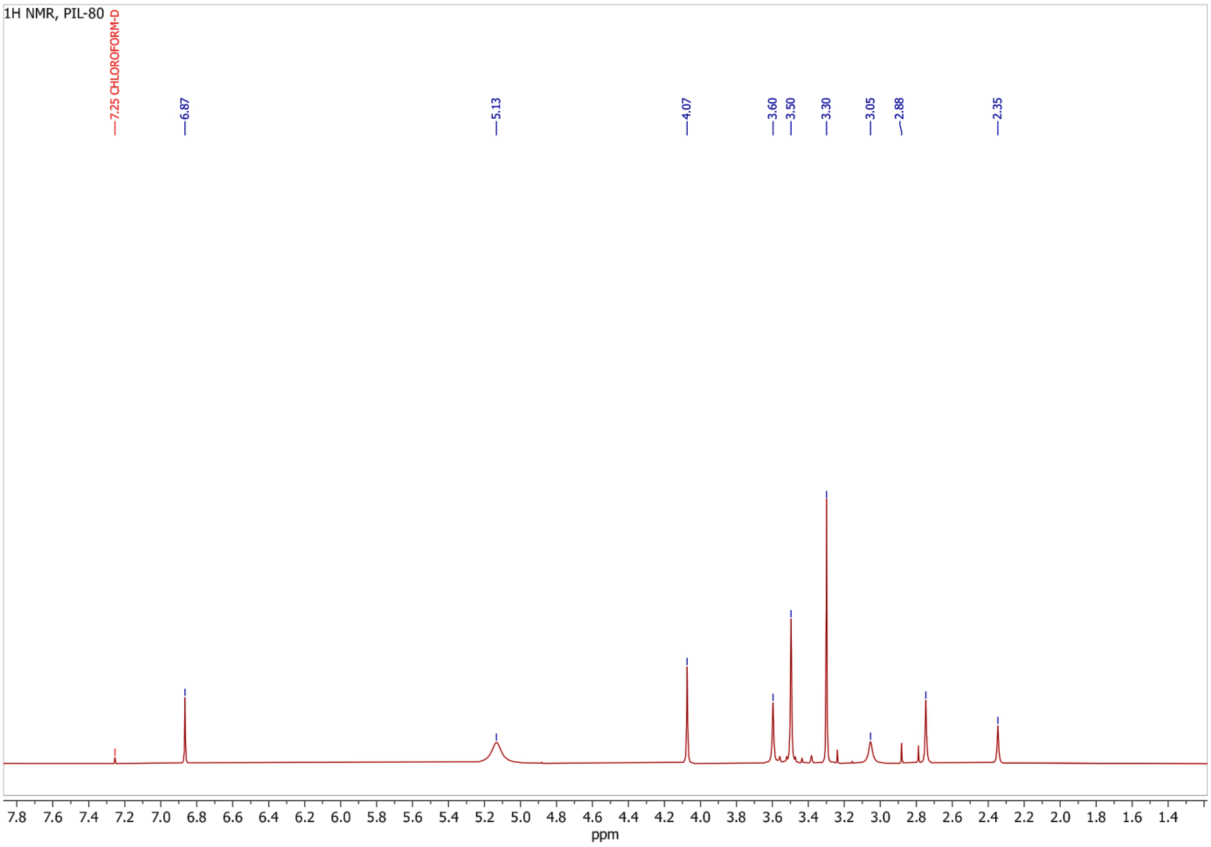


**Figure S7.**$^{1}$H NMR spectrum of PIL-80 (2) recorded in $CDCl_3$.

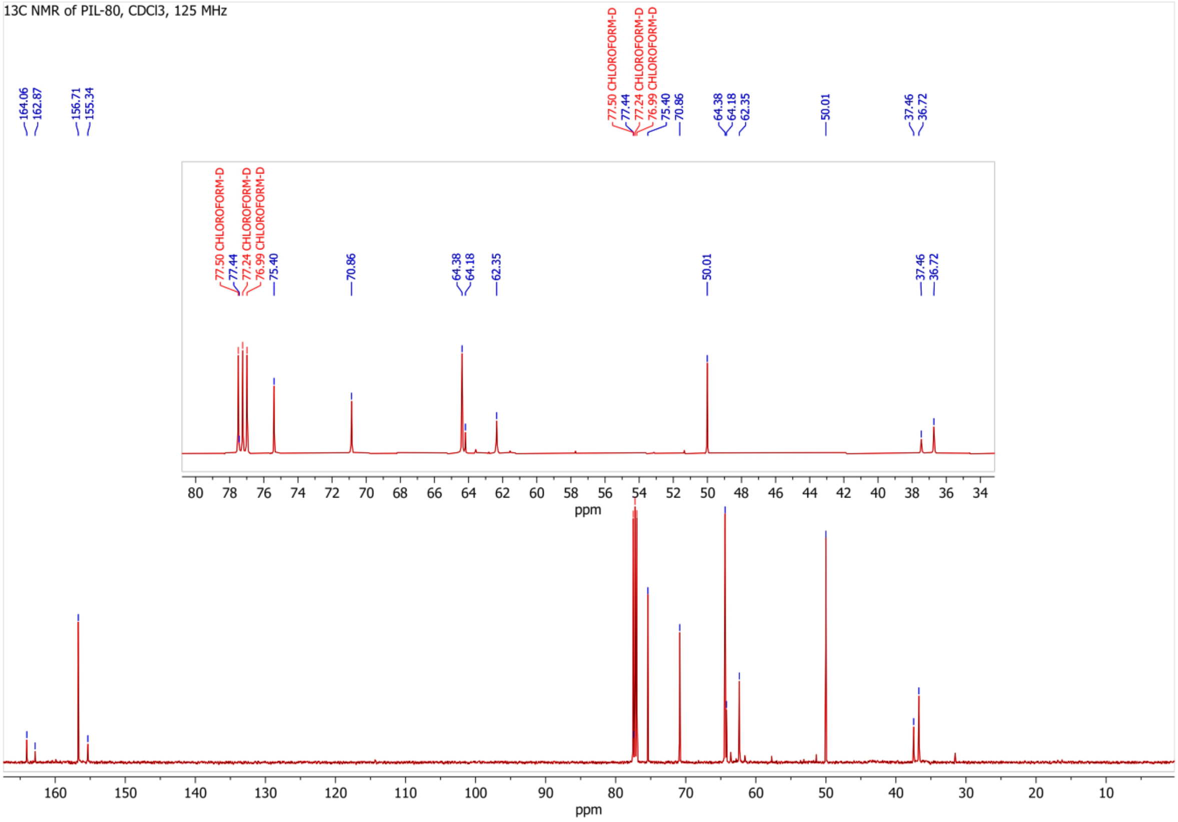


**Figure S8.**$^{13}$C NMR spectrum of PIL-80 (2) recorded in $CDCl_3$.
